# International Partnerships in Health Education: Adapting E-Learning Models for Conflict-Affected Myanmar

**DOI:** 10.3390/healthcare13030285

**Published:** 2025-01-31

**Authors:** Clelia D’Apice, Massimo Guasconi

**Affiliations:** 1 University Centre for International Cooperation, University of Parma, 43125 Parma, Italy; 2 Department of Medicine and Surgery, University of Parma, 43121 Parma, Italy; massimo.guasconi@unipr.it; 3 Azienda USL of Piacenza, 29121 Piacenza, Italy

**Keywords:** capacity building, conflict settings, E-learning, telesimulation, emergency medicine training, global health, healthcare education, international collaboration, sustainable development goals

## Abstract

Background: In the wake of Myanmar’s 2021 military coup, the University of Parma, in partnership with Myanmar and Brazilian institutions, developed an asynchronous e-learning program to sustain healthcare education amid severe disruptions. The program aimed to address urgent training needs in emergency medicine, public health management, and mental health, aligning with Sustainable Development Goals. Methods: An educational needs assessment involving 298 surveys and 10 interviews identified training priorities. Based on these findings, a four-module e-learning course was created, covering basic life support, trauma care, pediatric emergencies, and psychological assistance. The course utilized prerecorded high-fidelity telesimulations with multilingual support to ensure accessibility. Evaluation included participant satisfaction using the MSSE questionnaire and knowledge acquisition through post-module quizzes. Results: Over 750 students participated, with significant knowledge acquisition observed—60% scored 8 or higher across all modules. The MSSE questionnaire, completed by 152 students, revealed high satisfaction, with 88% agreeing that the course enhanced clinical reasoning, decision-making, and self-reflection Conclusions: This program demonstrates the value of international partnerships and e-learning in sustaining medical education during crises. High student engagement and strong learning outcomes affirm its efficacy. Future iterations will aim to improve completion rates, refine feedback mechanisms, and expand accessibility. This scalable model offers a blueprint for addressing healthcare training needs in conflict-affected and resource-limited settings, contributing to global health resilience and the achievement of Universal Health Coverage.

## 1. Introduction

The collaboration between the University of Parma (UNIPR) and Myanmar has played a critical role in strengthening healthcare systems by exchanging best practices in primary healthcare (PHC) and training for Universal Health Coverage (UHC). As the foundation of equitable and accessible healthcare, PHC is particularly crucial in resource-constrained settings. This partnership emphasizes capacity building in PHC as a key driver for achieving UHC, aligning closely with the Sustainable Development Goals (SDGs), particularly SDG 3: Good Health and Well-being [1,2,3].

Simultaneously, UNIPR has partnered with Brazilian institutions renowned for their robust PHC model, exemplified by Brazil’s Family Health Strategy [4]. This collaboration highlights the importance of leveraging international expertise to develop scalable and adaptable training programs that could address healthcare disparities and improve workforce capabilities in diverse settings [5,6].

Such efforts underscore the mutual benefits of the tripartite framework: Myanmar gains critical capacity-building support, Brazil refines its PHC strategies through adaptation, and UNIPR strengthens its role as a global health innovator. By fostering knowledge exchange and mutual learning, this partnership demonstrates the transformative power of international cooperation in addressing global health challenges, promoting equity, and building sustainable health systems [7,8,9,10].

The 2021 military coup in Myanmar triggered a severe political and humanitarian crisis, exacerbating systemic weaknesses in healthcare and education [11]. The healthcare system, already fragile due to years of underinvestment, has nearly collapsed. Hospitals have been attacked or repurposed for military use, while healthcare workers face violence, arrests, and persecution [11,12,13,14]. These conditions, combined with critical shortages of supplies and personnel, have left millions without access to essential care, increasing disease burdens and undermining public health [12,15,16,17].

The crisis has also severely impacted medical education and training. Universities and health education institutions are under military control and have been boycotted by students and faculty participating in the Civil Disobedience Movement (CDM) [14,18,19]. This disruption has created a vacuum in the training of future healthcare professionals. In response, students and educators have turned to alternative pathways, including online courses and community-led training initiatives, but these efforts face significant logistical and resource challenges [20,21]. Targeted violence against healthcare workers and institutions has not only deepened the immediate health crisis but also jeopardized the long-term resilience of the healthcare system [13,14,22]. The loss of trained professionals, coupled with the systematic dismantling of health infrastructure, threatens to create lasting gaps in healthcare delivery and education.

Despite these dire circumstances, healthcare workers and students have shown remarkable resilience. Many continue to resist through the CDM, supported by local communities and international organizations, while striving to sustain essential services and educational opportunities [14,18,19].

In this context, continuing UNIPR’s collaboration with Myanmar became both imperative and complex, raising a key question: how can international partnerships sustain capacity-building efforts in conflict and post-conflict settings?

To tackle these challenges, UNIPR prioritized online educational interventions that could be implemented despite the constraints of conflict. Research supports e-learning as an effective learning method for healthcare training, particularly during crisis when attending traditional in-person classes is unfeasible [23,24,25].

An educational needs assessment was conducted by UNIPR in collaboration with local stakeholders in Myanmar, consisting of a two-phase evaluation process. In the first phase, a comprehensive questionnaire was administered to Myanmar medical students, receiving responses from 298 participants to collect quantitative data on their perceived educational needs. The second phase involved qualitative interviews with 10 medical students, providing deeper insights into their experiences and highlighting specific areas where additional support was needed. The assessment identified three priority areas: emergency medicine, public health management, and mental health (an article detailing the implementation of the educational needs assessment is currently under publication). These domains were critical for responding to the immediate and long-term health needs arising from the crisis [26,27,28].

Building on these findings, UNIPR, in partnership with colleagues from the Laboratory for Technological Innovation in Health at the Federal University of Rio Grande do Norte (LAIS/UFRN), developed an online asynchronous training program tailored to the needs and realities of Myanmar’s healthcare workers and students (https://avasus.ufrn.br/local/avasplugin/cursos/curso.php?id=671 accessed on 30 November 2024). The course aimed to equip participants with life-saving skills, enhance their confidence in emergency response, and foster resilience in the face of ongoing challenges. Leveraging the Brazilian experience in PHC training [29], the program was designed to be practical, culturally sensitive, and easily replicable in other vulnerable situations.

This article aims to achieve two primary objectives:

1. To assess the satisfaction and perceived effectiveness of the online training program among participants.
2. To provide an example of how international collaboration in health education is crucial, particularly during emergencies such as conflicts, in sustaining healthcare training programs.

This research is significant as it addresses an urgent gap in healthcare education during crises, offering a model that can inform similar efforts in other conflict-affected or resource-constrained regions.

## 2. Materials and Methods

### 2.1. Online Course

Based on the educational needs assessment results, an online training program was designed consisting of four modules:

(i) “Introduction to Basic Life Support” (BLS): This module covers cardiopulmonary resuscitation (CPR) techniques, demonstrates and simulates the use of an automated external defibrillator (AED), and provides training on responding to potential choking situations. This module consists of 5 videos with a total runtime of approximately 20 min.
(ii) “Management of Trauma Care”: This module explains the essential characteristics of high-performing teams for assisting trauma patients in challenging environments, such as conflict zones. It includes instructions on the use of a tourniquet to control hemorrhages, the positioning of a pelvic stabilizer, and the performance of thoracostomy for pneumothorax management. This module consists of 5 videos with a total runtime of approximately 20 min.
(iii) “Pediatric emergency”: This module focuses on pediatric emergencies, emphasizing BLS, CPR, AED use, and managing special situations such as trauma, drowning, and choking. The benefits of telemedicine are also presented in this unit. The module includes 8 videos with a total runtime of approximately 1.5 h.
(iv) “Psychological assistance in emergencies”: This module examines psychological responses to emergencies, discusses psychological trauma and resilience, and introduces self-care techniques for emergency scenarios. It concludes with an overview of psychological first aid strategies. The module includes 5 videos with a total runtime of approximately 1.5 h.

Each module includes supporting materials for the videos in PDF format. They were developed using a combination of lectures and telesimulations.

Each module was developed using a combination of lectures and telesimulations. To ensure fluent delivery, the course was recorded in the trainers’ native languages (Italian and English). Additionally, English scripts and subtitles were prepared to accommodate Myanmar students, who spoke English.

### 2.2. Enrollment and Participants

The course was made available online in August 2024. We decided to conduct an evaluation of the first four months of activity. Participants were enrolled through convenience sampling; all students who accessed the course during the period under review were included. The sole inclusion criterion was being a Myanmar student in medicine, surgery, or other health professions.

The dissemination of the course was facilitated by both our team and our Brazilian colleagues, who shared the course link with professional contacts in Myanmar. These included networks of medical students involved in similar past initiatives, as well as community-based organizations (CBOs) and civil society organizations (CSOs). These groups and individuals further extended the course’s reach by sharing it within their networks. The course was designed to be freely accessible and open to all interested participants.

### 2.3. Data Collection and Analysis

Satisfaction levels were assessed using a questionnaire (MSSE—Appendix A), developed from the “Satisfaction With Simulation Experience” (SSE) scale [30] with the author’s permission. The original SSE scale comprises 18 items on a 5-point Likert scale (Strongly Disagree–Disagree–Unsure–Agree–Strongly Agree) divided into three areas:

(i) Debrief and Reflection, addressing the debriefing phase.
(ii) Clinical Reasoning, examining clinical reasoning skills.
(iii) Clinical Learning, evaluating satisfaction with clinical learning.

For this asynchronous e-learning context, the scale was adapted to nine items by excluding the debriefing-related area, as post-simulation debriefing was not feasible. Additionally, the term “simulation” was replaced with “telesimulation”.

The questionnaire was integrated into the course platform. Students who completed all modules could voluntarily complete the MSSE.

The training’s effectiveness was assessed using multiple-choice quizzes. The quiz questions were created by the instructor of each module, based on the training content. Each module included five questions, each with four possible answers, of which only one was correct. These quizzes were administered at the end of each module.

Quiz and MSSE data were downloaded anonymously and in aggregate form, ensuring that individual participants remained unidentifiable. Data were analyzed using Microsoft Excel 365. Agreement percentages for MSSE items and correct response percentages for end-of-module quizzes were calculated. To provide students with numerical feedback on the unit quizzes, two points were awarded for each correct answer. Since each unit contained five questions, total scores ranged from 0 (no correct answers) to 10 (all correct answers). Median and interquartile range were calculated for total quiz scores.

### 2.4. Ethical Considerations

This study adhered to the European Code of Conduct for Research Integrity, the principles of the Declaration of Helsinki, and the Good Clinical Practice guidelines. The Research Ethics Board (REB) of the University of Parma approved the study on 6 March 2024, under protocol 0075678.

## 3. Results

As of November 22, 2024, Unit 1 was completed by 754 students, Unit 2 by 660, Unit 3 by 632, and Unit 4 by 652 students.

For the evaluation quiz in Unit 1, the percentage of correct answers was as follows: 86.34% for the first question (Q1), 85.01% for the second question (Q2), 68.57% for the third question (Q3), 55.44% for the fourth question (Q4), and 68.17% for the fifth and final question (Q5) (Table 1). Regarding the total scores achieved by the participants, 33.29% scored 10 points, 28.91% scored 8 points, 20.16% scored 6 points, 7.29% scored 4 points, 6.37% scored 2 points, and the remaining 3.98% scored 0 points (Table 1). The median score for Unit 1 was 8 (6–10).

In Unit 2, the percentage of correct answers for each question was as follows: 81.67% for Q1, 76.67% for Q2, 66.21% for Q3, 78.18% for Q4, and 86.52% for Q5 (Table 2). Regarding the total scores achieved by the participants, 50.76% scored 10 points, 17.27% scored 8 points, 13.94% scored 6 points, 9.39% scored 4 points, 5.76% scored 2 points, and the remaining 2.88% scored 0 points (Table 2). The median score for Unit 2 was 10 (6–10).

In Unit 3, the percentage of correct answers for each question was as follows: 84.81% for Q1, 70.09% for Q2, 79.27% for Q3, 59.49% for Q4, and 84.18% for Q5 (Table 3). Regarding the total scores achieved by the participants, 40.82% scored 10 points, 28.96% scored 8 points, 11.55% scored 6 points, 7.91% scored 4 points, 7.44% scored 2 points, and the remaining 3.32% scored 0 points (Table 3). The median score for Unit 3 was 8 (6–10).

In Unit 4, the percentage of correct answers for each question was as follows: 72.61% for Q1, 69.17% for Q2, 75.00% for Q3, 83.28% for Q4, and 84.97% for Q5 (Table 4). Regarding the total scores achieved by the participants, 55.21% scored 10 points, 14.57% scored 8 points, 10.43% scored 6 points, 5.52% scored 4 points, 7.98% scored 2 points, and the remaining 6.29% scored 0 points (Table 4). The median score for Unit 4 was 10 (6–10).

Student satisfaction was assessed using the MSSE scale, completed by 152 students. The results are summarized in Table 5.

As shown in the table, most students reported being satisfied with their learning experience.

## 4. Discussion

Health systems mostly depend on local educational structures to facilitate the training of an adequate supply of health professionals. Inadequate levels of physicians are associated with increased population disease burden and a reduction in health system performance [17,31]. In this context, international cooperation can play a pivotal role in addressing these gaps [27,32]. Building on the longstanding collaboration between the University of Parma, Brazil, and Myanmar, we decided to support medical education in Myanmar through the implementation of an online training program.

In emergency contexts, including conflict-affected areas, online education offers a vital opportunity to ensure continuity in healthcare training [33,34]. E-learning has been widely recognized as a flexible and effective approach to overcome barriers such as geographical isolation, limited infrastructure, and security risks [35,36]. Research highlights its capacity to deliver high-quality instruction, even in crises, allowing learners to access resources and acquire skills remotely [23,24,25,37]. Moreover, several studies are also demonstrating the value of telesimulation in conflict zones, enabling practical skill development through remote, interactive training methods [38,39,40].

This initiative aims to contribute to the medical education of Myanmar medical students and health professionals who are unable to attend medical schools due to the ongoing conflict. We firstly conducted an educational needs assessment of Myanmar medical students, and we then developed, in partnership with colleagues from LAIS/URFN, an online asynchronous training program tailored to the needs and realities emerging from the assessment.

This article seeks to evaluate both the satisfaction and perceived effectiveness of the online training program among participants, while also demonstrating the critical role of international collaboration in sustaining healthcare training programs, especially during emergencies like conflicts.

The significant participation rates for the online course, with 754 students completing the most attended unit and 632 students completing the least attended, demonstrate the feasibility and adaptability of such programs in resource-constrained and conflict-affected settings [23]. However, the variation in completion rates across modules warrants closer examination. Differences in participation may stem from the sequential nature of the course, as not all students may have completed all units by the time of data collection. Additional factors include connectivity challenges, which are well-documented barriers in online education within conflict zones [41,42]. Despite Myanmar students reporting secure internet access during the needs assessment, intermittent disruptions likely affected participation. Further, declining motivation over time, a known issue in online learning [43], could also have contributed.

Importantly, Myanmar medical students face unique difficulties due to the civil war, including disruptions to formal education and active involvement in medical responses to the conflict [44,45]. This study provides a unique perspective, as it captures the students’ experiences during the conflict, rather than retrospectively, offering critical insights into the evolving challenges of medical training in such conditions. Future iterations of this program could integrate strategies to mitigate these challenges, such as offering offline-accessible modules, flexible pacing, and periodic motivational incentives to sustain engagement.

The quiz results confirm the program’s effectiveness in imparting essential knowledge and skills, with over 60% of participants scoring 8 or higher across all units. This is confirmed by median scores of 8 (6–10) for Units 1 and 3, and 10 (6–10) for Units 2 and 4. These findings align with previous studies on the efficacy of e-learning in healthcare education [46,47,48]. Telesimulation is increasingly employed in healthcare education, typically involving real-time online interaction between instructors and students [24,49,50]. However, this study’s use of pre-recorded high-fidelity simulations presents an innovative alternative, tailored to the logistical constraints of conflict-affected regions [51]. By allowing students to follow step-by-step demonstrations independently, this approach minimizes reliance on stable internet connectivity while maintaining educational rigor.

The MSSE scale results provide additional insight into student satisfaction. With over 88% agreeing or strongly agreeing to all items, the program effectively enhanced clinical reasoning, decision-making, and self-reflection. These outcomes align with findings from Gerstenberger [52] on the educational value of telesimulation. However, the limited response rate to the MSSE survey, relative to total enrollment, indicates an area for improvement in future iterations. Incorporating incentivized feedback mechanisms or integrating evaluations directly into course completion criteria could enhance response rates and provide richer data for program refinement. Additionally, the absence of synchronous debriefing sessions, a key component of traditional telesimulation [50], may have limited opportunities for reflection and peer learning. Alternatives, such as asynchronous discussion forums or guided self-assessment modules, could address this limitation and enhance the learning experience.

The overall success of this program is deeply rooted in the collaborative framework that guided its development and implementation. The partnership between the University of Parma, Myanmar stakeholders, and Brazilian institutions demonstrates the transformative potential of international cooperation in addressing global health challenges. Collaboration between countries with different healthcare realities allows for the sharing of diverse perspectives and expertise, fostering innovative approaches to complex problems. International academic collaborations are instrumental in addressing global healthcare challenges, enabling the exchange of knowledge and fostering innovation in medical education across diverse contexts, including resource-limited and crisis-affected regions [53,54]. Brazil’s Family Health Strategy, for instance, served as a foundation for designing modules tailored to primary healthcare needs in conflict zones, highlighting the mutual benefits of adapting proven strategies to new contexts [4,29].

Such cross-cultural knowledge exchange not only fosters innovation but also reinforces global solidarity—a critical element during crises.

Myanmar’s healthcare system, already strained before the 2021 military coup, faced severe near-total collapse during the ongoing conflict [11,15]. In this context, international academic partnerships play a vital role in sustaining educational continuity and addressing immediate healthcare workforce needs [55,56]. This study demonstrates how e-learning can serve as a critical lifeline for medical education, enabling the delivery of essential training even under the most challenging circumstances [3,7].

Moreover, collaboration fosters mutual learning and capacity enhancement. While Myanmar benefits from targeted training, Brazilian and Italian partners gain insights into the challenges of healthcare delivery in crisis settings, which can inform future innovations in their own contexts. This bidirectional exchange not only enhances the program’s impact but also strengthens the participating institutions’ global health capabilities. The program exemplifies how shared objectives, pooled expertise, and mutual respect can create sustainable solutions that transcend geographical and political boundaries.

The broader implications of this program extend well beyond Myanmar, offering a scalable and adaptable model for healthcare education in low-resource or crisis-affected regions. This approach has garnered interest from health professionals and researchers working in conflict-impacted areas, highlighting its potential to address global challenges in medical training and education under adverse conditions [27,31,57]. By leveraging innovative methodologies and context-sensitive adaptations [58], such initiatives can serve as blueprints for similar interventions in other regions facing comparable constraints. However, replicating the program in different regions requires careful evaluation of local conditions, including infrastructure healthcare challenges, and digital literacy. Key requirements for successful implementation include stable internet access, which is essential for the program’s success. Given the program’s self-access nature, it also needs to be evaluated for feasibility in regions with limited technological infrastructure.

Its design aligns with global calls for innovative strategies to achieve Universal Health Coverage and the Sustainable Development Goals, particularly SDG 3: Good Health and Well-being [3]. As such, this program contributes to addressing immediate training needs while building a foundation for long-term capacity development and health system resilience [59,60]. By addressing immediate training needs and laying the groundwork for long-term health system resilience, this program exemplifies the critical role of adaptable, collaborative approaches in global health education.

### Limitations

This study inevitably has certain limitations: (i) The socio-political conditions in Myanmar prevented the collection of students’ socio-demographic data, thus limiting the ability to further analyze the results obtained. (ii) For the same reason, the MSSE data were provided by the AVASUS platform in an aggregated form, making it impossible to perform inferential statistical analyses, such as calculating Cronbach’s alpha, to assess its validity. The validity of the MSSE should be tested in the future.

## 5. Conclusions

This initiative exemplifies the transformative power of international partnerships in addressing global health challenges. The collaboration between the University of Parma, Myanmar institutions, and Brazilian partners has led to the development of an innovative and adaptable online training program. By leveraging e-learning and telesimulation, the program has effectively addressed urgent healthcare training needs in Myanmar, providing critical support in emergency medicine, public health management, and mental health during a time of profound crisis.

The program’s success underscores the importance of mutual learning, capacity-building, and the ability to innovate in healthcare education, particularly during emergencies. High levels of student satisfaction and strong performance in knowledge assessments demonstrate the program’s effectiveness in enhancing clinical reasoning, decision-making, and preparedness for emergency scenarios. These findings suggest that similar approaches could be adapted for other conflict-affected or resource-constrained settings, with careful tailoring to local needs.

Moving forward, enhancing the feedback mechanisms and exploring hybrid learning models could further improve the program’s impact and long-term sustainability. This collaboration also reinforces the critical role of tailored educational interventions in meeting the specific and context-related needs of conflict-affected populations. While the program is adaptable, its replicability in other regions requires careful modifications to suit the specific challenges and infrastructure of each setting.

In the future, e-learning programs could be integrated with telemedicine programs and their effectiveness evaluated.

This project serves as a model for future international initiatives aimed at strengthening healthcare systems through innovative educational strategies, contributing to long-term health system strengthening in crisis-affected areas. Further, it underscores the importance of Universal Health Coverage and Sustainable Development Goal 3 as guiding frameworks for global health education and policy alignment, particularly in emergency settings.

## Figures and Tables

**Table 1:** Results of the evaluation quiz for Unit 1 (*n* = 754).

UNIT 1						
How should chest compressions be performed during CPR?		Score		*n*	%	
		0		101	13.40%	
		2		651	86.34%	
		-		2	0.27%	
		Tot.		754	100.00%	
What should you do when the AED recommends a shock?		Score				
		0		109	14.46%	
		2		641	85.01%	
		-		4	0.53%	
		Tot.		754	100.00%	
What is the role of CPR (Cardiopulmonary Resuscitation) immediately after successful defibrillation?		Score				
		0		235	31.17%	
		2		517	68.57%	
		-		2	0.27%	
		Tot.		754	100.00%	
What is the correct sequence for performing the Heimlich manoeuvre in the infant patient?		Score				
		0		332	44.03%	
		2		418	55.44%	
		-		4	0.53%	
		Tot.		754	100.00%	
Which technique is described for performing chest compressions in infants?		Score				
		0		239	31.70%	
		2		514	68.17%	
		-		1	0.13%	
		Tot.		754	100.00%	
		Total score				
		0		30	3.98%	
		2		48	6.37%	
		4		55	7.29%	
		6		152	20.16%	
		8		218	28.91%	
		10		251	33.29%	
		Tot.		754	100.00%	

**Table 2:** Results of the evaluation quiz for Unit 2 (*n* = 660).

UNIT 2			
What is the first step in immobilizing the cervical spine for the suspected traumatized patient?	Score	*n*	%
	0	121	18.33%
	2	539	81.67%
	-	0	0%
	Tot.	660	100.00%
What are the key steps to ensure correct and effective positioning of a tourniquet?	Score		
	0	151	22.88%
	2	506	76.67%
	-	3	0.45%
	Tot.	660	100.00%
What does scientific evidence suggest regarding the safety of tourniquet application?	Score		
	0	219	33.18%
	2	437	66.21%
	-	4	0.61%
	Tot.	660	100.00%
What is the primary purpose of placing a needle inside the medullary venous plexus of the spongy bone tissue?	Score		
	0	141	21.36%
	2	516	78.18%
	-	3	0.45%
	Tot.	660	100.00%
According to the modified Scott’s Algorithm, what are the indications for the positioning of the pelvic stabilizer?	Score		
	0	89	13.48%
	2	571	86.52%
	Tot.	660	100.00%
	Total Score		
	0	19	2.88%
	2	38	5.76%
	4	62	9.39%
	6	92	13.94%
	8	114	17.27%
	10	335	50.76%
	Tot.	660	100.00%

**Table 3:** Results of the evaluation quiz for Unit 3 (*n* = 632).

UNIT 3			
Why is it crucial to remove wet clothing and thoroughly dry a drowned child before applying defibrillation pads?	Score	*n*	%
	0	95	15.03%
	2	536	84.81%
	-	1	0.16%
	Tot.	632	100.00%
What is the recommended initial action when a child experiences a partial airway obstruction by a food item and is conscious and coughing?	Score		
	0	187	29.59%
	2	443	70.09%
	-	2	0.32%
	Tot.	632	100.00%
What should be done if a child with a food-induced complete airway obstruction becomes cyanotic and unresponsive but is still conscious?	Score		
	0	131	20.73%
	2	501	79.27%
	Tot.	632	100.00%
In pediatric Basic Life Support (BLS), how does the compression-to-ventilation ratio and technique vary between a single rescuer and two rescuers for infants?	Score		
	0	254	40.19%
	2	376	59.49%
	-	2	0.32%
	Tot.	632	100.00%
In the management of a 6-year-old child who suffered a fall from a height of 1.5 m, why was an initial assessment following the ABCDE protocol critical?	Score		
	0	99	15.66%
	2	532	84.18%
	-	1	0.16%
	Tot.	632	100.00%
	Total Score		
	0	21	3.32%
	2	47	7.44%
	4	50	7.91%
	6	73	11.55%
	8	183	28.96%
	10	258	40.82%
	Tot.	632	100.00%

**Table 4:** Results of the evaluation quiz for Unit 4 (*n* = 652).

UNIT 4			
When the “freeze” response or “shutdown” occurs, we may feel:	Score	*n*	%
	0	180	27.61%
	2	471	72.24%
	-	1	0.15%
	Tot.	652	100.00%
The following symptoms category is NOT included in four main categories of Post-Traumatic Stress Disorder (PTSD).	Score		
	0	200	30.67%
	2	451	69.17%
	-	1	0.15%
	Tot.	652	100.00%
If we experience the following condition, we can still manage our distress by ourselves and there is no need to reach out to mental health professionals for support.	Score		
	0	161	24.69%
	2	489	75.00%
	-	2	0.31%
	Tot.	652	100.00%
Psychological first aid (PFA) can be provided by:	Score		
	0	107	16.41%
	2	543	83.28%
	-	2	0.31%
	Tot.	652	100.00%
When we are providing Psychological First Aid (PFA), we should:	Score		
	0	98	15.03%
	2	554	84.97%
	Tot.	652	100.00%
	Total Score		
	0	41	6.29%
	2	52	7.98%
	4	36	5.52%
	6	68	10.43%
	8	95	14.57%
	10	360	55.21%
	Tot.	652	100.00%

**Table 5:** Results of the MSSE scale (*n* = 152).

		Strongly Disagree	Disagree	Unsure	Agree	Strongly Agree	Tot.
The telesimulation developed my clinical reasoning skills	*n*	6	2	9	63	71	151
	%	4	1	6	42	47	100
The telesimulation developed my clinical decision making ability	*n*	3	3	8	65	72	151
	%	2	2	5	43	48	100
The telesimulation enabled me to demonstrate my clinical reasoning skills	*n*	4	4	8	56	76	148
	%	3	3	5	38	51	100
The telesimulation helped me to recognise patient deterioration early	*n*	3	2	9	61	76	151
	%	2	1	6	40	50	1
This was a valuable learning experience	*n*	4	3	3	47	95	152
	%	3	2	2	31	63	100
The telesimulation caused me to reflect on my clinical ability	*n*	5	3	11	62	70	151
	%	3	2	7	41	46	100
The telesimulation tested my clinical ability	*n*	2	3	16	60	70	151
	%	1	2	11	40	46	100
The telesimulation helped me to apply what I learned from the case study	*n*	2	3	12	62	72	151
	%	1	2	8	41	48	100
The telesimulation helped me to recognise my clinical strengths and weaknesses	*n*	3	4	12	57	76	152
	%	2	3	8	38	50	100

## Data Availability

The raw data supporting the conclusions of this article will be made available by the authors on request.

## Appendix A. Modified Satisfaction with Simulation Experience (MSSE) Scale

In the next pages, you will find a list of statements. Read each statement and then select the response that best indicates your level of agreement.

- Please answer every item, even if one seems similar to another one
- Answer each item quickly, without spending too much time on any one item.

**Clinical reasoning**		
10	The telesimulation developed my clinical reasoning skills	Strongly disagree→Disagree→Unsure Agree→Strongly agree
11	The telesimulation developed my clinical decision making ability	Strongly disagree→Disagree→Unsure Agree→Strongly agree
12	The telesimulation enabled me to demonstrate my clinical reasoning skills	Strongly disagree→Disagree→Unsure Agree→Strongly agree
13	The telesimulation helped me to recognise patient deterioration early	Strongly disagree→Disagree→Unsure Agree→Strongly agree
14	This was a valuable learning experience	Strongly disagree→Disagree→Unsure Agree→Strongly agree
**Clinical learning**		
15	The telesimulation caused me to reflect on my clinical ability	Strongly disagree→Disagree→Unsure Agree→Strongly agree
16	The telesimulation tested my clinical ability	Strongly disagree→Disagree→Unsure Agree→Strongly agree
17	The telesimulation helped me to apply what I learned from the case study	Strongly disagree→Disagree→Unsure Agree→Strongly agree
18	The telesimulation helped me to recognise my clinical strengths and weaknesses	Strongly disagree→Disagree→Unsure Agree→Strongly agree
This instrument was derived from [30]		
